# The Role of Artificial Intelligence in the Diagnosis, Segmentation, and Prediction of Retinal Vein Occlusion: A Systematic Review

**DOI:** 10.7759/cureus.97419

**Published:** 2025-11-21

**Authors:** Eirini Maliagkani, Vyron Michalakis, Ioannis D Apostolopoulos, Konstantinos Tyrlis, Ilias Georgalas

**Affiliations:** 1 First Department of Ophthalmology, General Hospital of Athens "G. Gennimatas" Medical School of the National and Kapodistrian University of Athens, Athens, GRC; 2 Applied Computing and Technological Applications (ΑCTA) Lab, University of Thessaly, Larisa, GRC

**Keywords:** artificial intelligence, deep learning, diagnosis, ophthalmology, prediction, retinal imaging, retinal vein occlusion, segmentation

## Abstract

Retinal vein occlusion (RVO) is the second most common cause of vision loss after diabetic retinopathy. It results from the occlusion of either the central retinal vein or one of its branches. Artificial intelligence (AI), particularly deep learning (DL), has shown great potential in ophthalmology for disease assessment. This review examined how AI has been applied to the diagnosis, segmentation, and treatment prediction of RVO across different imaging modalities.

A comprehensive search of PubMed, Scopus, and Google Scholar up to June 19, 2024, identified 2,925 records, of which 23 met the inclusion criteria. Most studies (91%) were published after 2020, reflecting the rapid growth of AI in this field. DL algorithms were used in 87% of studies, mainly convolutional neural networks such as Residual Network, Densely Connected Convolutional Network, and Visual Geometry Group Network. Classification was the most frequent task (78%), followed by segmentation (26%) and prediction (17%). Color fundus photography was the most common imaging modality (57%), followed by fluorescein angiography (26%), with fewer studies using optical coherence tomography or optical coherence tomography angiography.

Internal validation metrics were generally high (accuracy 0.79-0.99, sensitivity 0.67-1.00, specificity 0.80-1.00), but performance declined in external validation (accuracy 0.39-0.98, sensitivity 0.38-0.93), indicating limited generalizability. Segmentation models achieved Dice coefficients between 0.82 and 0.94. Only 30% of studies used external datasets, and one performed clinical validation. Explainable AI techniques were applied in 39% of studies, mostly Grad-CAM, though often in a qualitative manner.

Overall, AI systems demonstrate strong potential for assisting in RVO diagnosis and management, but challenges remain. Limited dataset diversity, lack of multimodal fusion, and minimal clinical validation restrict real-world applicability. Future research should prioritize multicenter datasets, standardized evaluation, interpretability, and ethical governance to enable safe and effective integration of AI tools in ophthalmic care.

## Introduction and background

Retinal vein occlusion (RVO) is a vascular retinal disease caused by either central retinal vein occlusion (CRVO) or branch retinal vein occlusion (BRVO), which can result in vision impairment and long-term complications. RVO, including central (CRVO) and branch (BRVO) types, is a major cause of visual loss, particularly in patients with vascular comorbidities such as hypertension and diabetes. RVO is the second most common cause of vision loss after diabetic retinopathy, affecting an estimated 16.4 million people, the majority of whom have BRVO. No significant sex differences in incidence have been reported [1].

Artificial intelligence (AI) has become increasingly integrated into ophthalmic research, offering new possibilities for the detection, segmentation, and prediction of retinal diseases such as RVO. Deep learning (DL), a subset of machine learning (ML), has shown remarkable potential in analyzing medical images by automatically identifying complex patterns that may not be evident to clinicians. Among DL architectures, convolutional neural networks (CNNs) are the most widely used for image-based tasks. These models can efficiently extract spatial and structural features from retinal images, enabling automated identification of vascular changes, hemorrhages, and macular edema associated with RVO [2-5].

In ophthalmology, CNN-based systems have been successfully applied across imaging modalities, including fundus photography, optical coherence tomography (OCT), and fluorescein angiography (FA), facilitating diagnostic classification, lesion segmentation, and prognostic modeling. Transfer learning and model fine-tuning have further improved performance, allowing adaptation of pretrained networks to RVO-specific datasets even when data availability is limited [6-10].

Despite these advances, challenges remain regarding model interpretability, generalizability, and the need for external validation across diverse populations and imaging devices. Furthermore, ethical and regulatory considerations continue to shape the clinical translation of AI models [11-13].

The purpose of this review is to examine how AI has been utilized in the diagnosis (classification), segmentation, and prediction of RVO, highlighting current applications, performance, and existing limitations across various imaging modalities.

## Review

Materials and methods

Search Strategy

A comprehensive literature search was conducted in the PubMed, Elsevier, and Google Scholar databases up to June 19, 2024. The following Boolean search string was used in PubMed and adapted as appropriate for the other databases: (“artificial intelligence” OR “machine learning” OR “deep learning” OR “neural networks” OR “convolutional neural networks” OR “automated diagnosis” OR “automated screening”) AND (“retinal vein occlusion” OR “branch retinal vein occlusion” OR “central retinal vein occlusion” OR “RVO” OR “BRVO” OR “CRVO”).

Only English-language articles were included, and no geographic restrictions were applied. Reference lists of the included articles were manually screened to identify additional eligible studies. All retrieved records were imported into Mendeley Reference Manager (Mendeley Ltd., London, United Kingdom), where duplicates were removed and titles/abstracts were screened for relevance. Two independent authors (E.M. and V.M.) screened all titles and abstracts for eligibility, with discrepancies resolved by consensus after discussion. Full-text screening was also performed independently by the same authors, and any disagreements were resolved by a third author (I.D.A).

Eligibility Criteria

Studies were included in this review if they applied AI methods, including ML or DL, to diagnose, classify, segment, detect, or predict RVO. Both CRVO and BRVO subtypes were eligible. Studies had to use ophthalmic imaging techniques such as fundus photography, OCT, OCT angiography (OCTA), FA, or other similar retinal imaging modalities. Only studies that used a total dataset of at least 100 images and reported quantitative AI performance measures (for example, accuracy, sensitivity, or specificity) were included. Furthermore, only full-text articles published in English were considered.

Studies were excluded if they did not involve RVO cases, did not apply AI techniques, or used datasets with fewer than 100 images. Studies were also excluded if they lacked quantitative performance metrics or statistical evaluation of AI results, involved nonhuman subjects, or were published as reviews, case reports, conference abstracts, editorials, or presentations. Articles without an accessible full text or not published in English were also excluded from the review.

Data Extraction

Data were extracted using a predefined, customized Microsoft Excel spreadsheet (Microsoft Corporation, Redmond, WA), which included the following fields: author, year, disease type, dataset (name and origin), imaging modality, total images, AI task, AI type, AI architecture, explainable AI (XAI) technique, external validation, clinical validation, AI model performance (internal), AI model performance (external), and AI model performance (clinical). Data extraction was performed independently by two authors (E.M. and V.M.) to ensure accuracy and consistency. Any discrepancies or uncertainties were discussed and resolved by consensus, with arbitration by a third author (I.D.A.) when required. Extracted data were reviewed collaboratively before synthesis to ensure completeness and consistency.

Results

Study Selection

**Figure 1:**
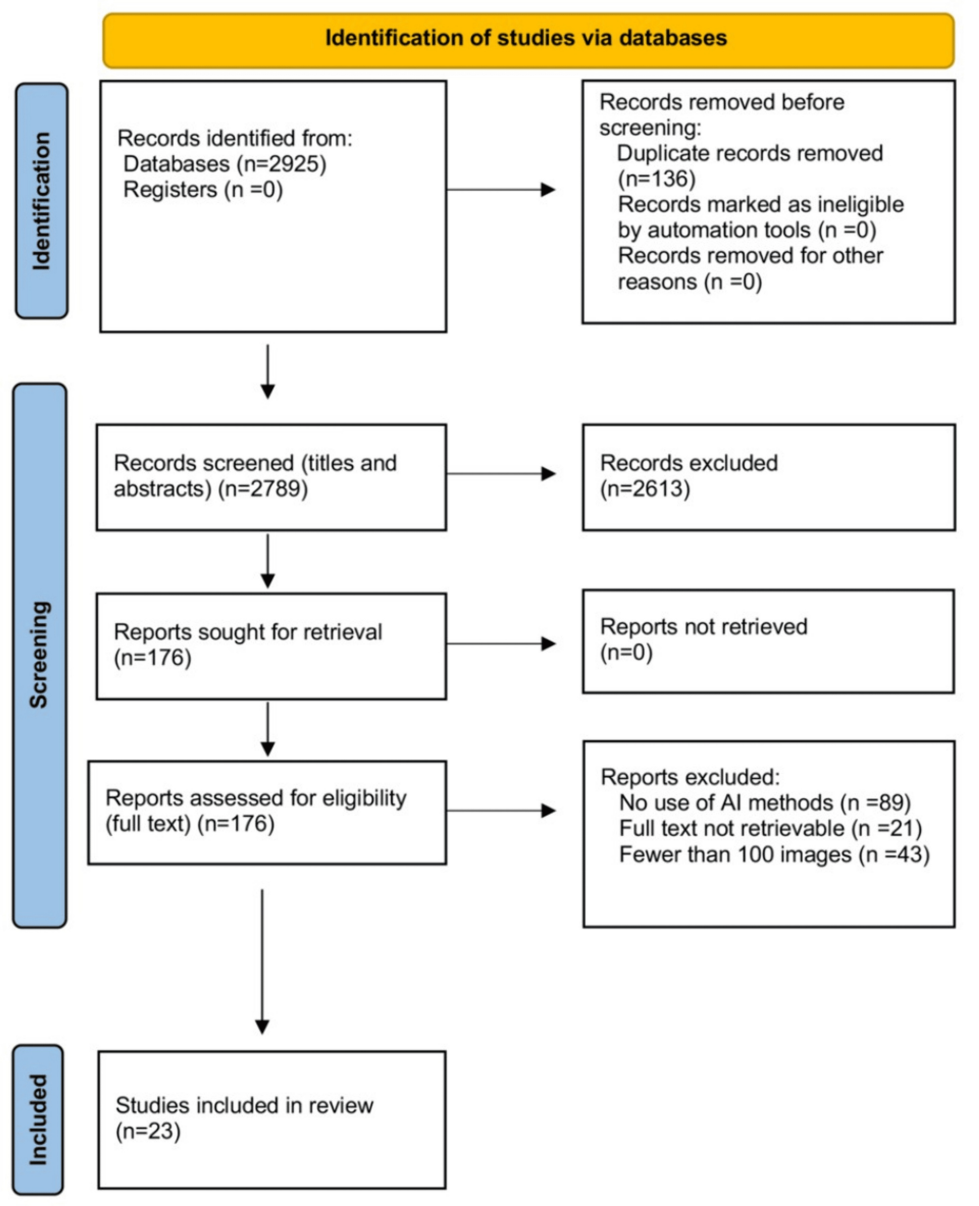
PRISMA 2020 flowchart PRISMA: Preferred Reporting Items for Systematic Reviews and Meta-Analyses

The initial literature search yielded 2,925 studies. A detailed visual representation of the study selection process is provided  in the Preferred Reporting Items for Systematic Reviews and Meta-Analyses flowchart (Figure 1) [14]. After duplicate removal in Mendeley, 2,789 studies remained for title and abstract screening, leading to the exclusion of 2,613 records, mainly due to an ineligible population (e.g., studies conducted in individuals without RVO) or an ineligible intervention (e.g., RVO analysis without the use of AI tools). A total of 176 full-text articles were assessed for eligibility. Of these, 153 were excluded for the following reasons: no use of AI methods (n = 89), full text not retrievable (n = 21), or datasets containing fewer than 100 images (n = 43). Finally, 23 studies met all eligibility criteria and were included in the qualitative synthesis [15-37].

Characteristics of Included Studies

**Figure 2:**
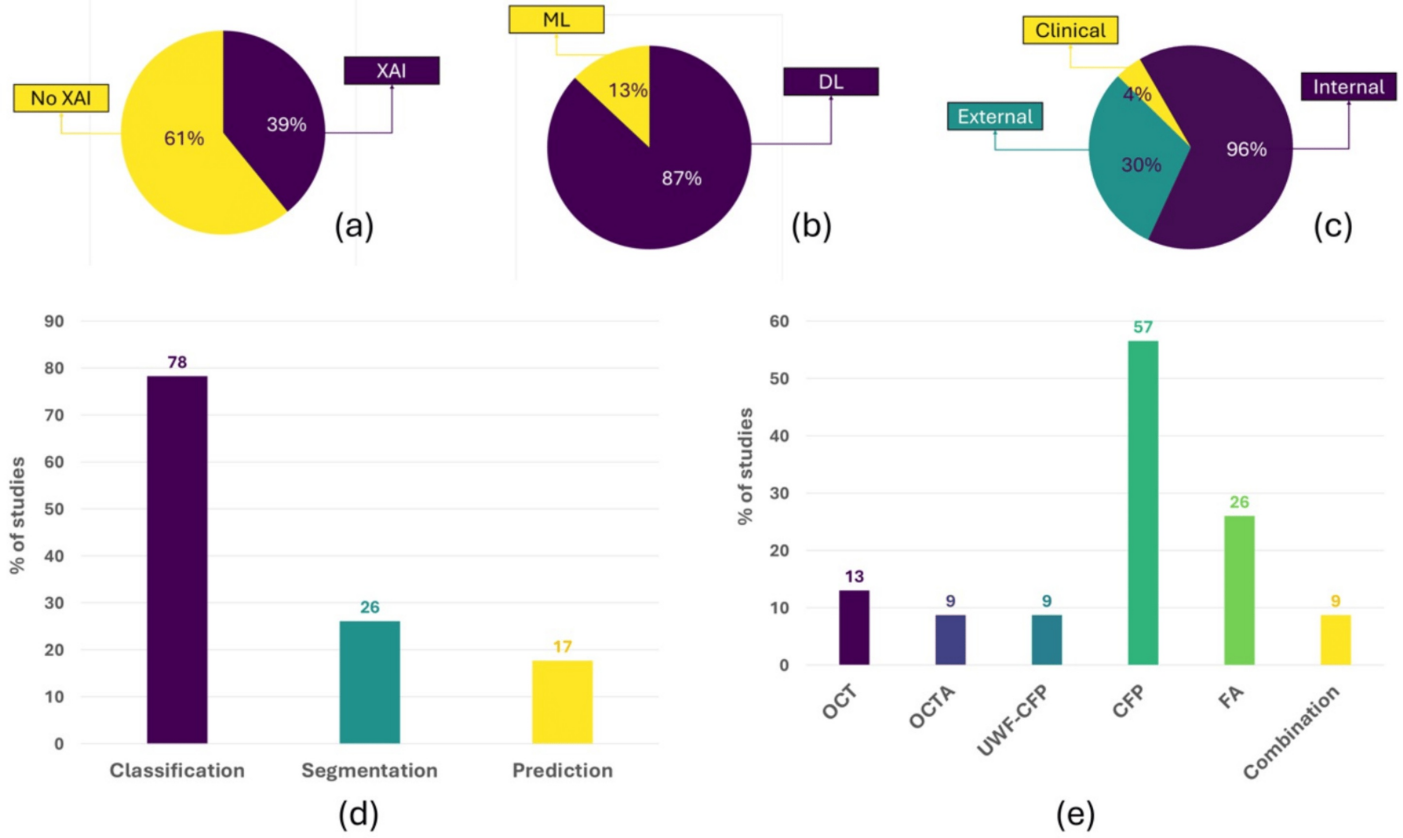
Overview of artificial intelligence applications in retinal vein occlusion studies. (a) Proportion of studies that used XAI vs. those without XAI. (b) Distribution of traditional ML and DL approaches. (c) Proportion of studies using internal, external, or clinical validation. (d) Distribution of classification, segmentation, and prediction AI tasks. (e) Imaging modalities used across studies include OCT, OCTA, UWF-CFP, CFP, FA, and multimodal combinations XAI: explainable artificial intelligence, ML: machine learning, DL: deep learning, OCT: optical coherence tomography, OCTA: optical coherence tomography angiography, UWF-CFP: ultrawide-field color fundus photograph, CFP: color fundus photograph, FA: fluorescein angiography

Across the included studies (Figure 2 and Tables 1, 2), AI was used primarily for classification (78%), followed by segmentation (26%) and treatment prediction (17%). Some studies addressed multiple tasks: 26% examined both classification and segmentation, and one study covered classification and prediction [23]. No studies have investigated segmentation and prediction together, nor have any examined classification, segmentation, and prediction jointly.

**Table 1:** Study and model characteristics AI: artificial intelligence, anti-VEGF: antivascular endothelial growth factor, BRVO: branch retinal vein occlusion, CAM: class activation mapping, CFP: color fundus photography, class.: classification, CRVO: central retinal vein occlusion, DL: deep learning, DME: diabetic macular edema, DR: diabetic retinopathy, FA: fluorescein angiography, FSPH: Foshan Second People's Hospital, GAN: generative adversarial network, Grad-CAM: gradient-weighted class activation mapping, GVM: graph visualization map, HLBP: hierarchical local binary pattern, ICG: indocyanine green angiography, MBRVO: macular branch retinal vein occlusion, mCNV: myopic choroidal neovascularization, ME: macular edema, ML: machine learning, MRVO: macular retinal vein occlusion, nAMD: neovascular age-related macular degeneration, NUN: neural understanding network, OCT: optical coherence tomography, OCTA: optical coherence tomography angiography, PR: private, pred.: prediction, PU: public, RVO: retinal vein occlusion, SCR: sickle cell retinopathy, segm.: segmentation, SEH: Shenzen Eye Hospital, SVM: support vector machine, UWF-CFP: ultra-wide-field color fundus photography, XAI: explainable artificial intelligence

Study	Disease type	Dataset	Imaging modality	Total images	AI task	AI type	ΑΙ architecture	XAI	External validation	Clinical validation
Abitbol et al. [15]	RVO; DR; SCR; healthy	Creteil University Hospital, France (PR)	UWF-CFP	224	Classification	DL	DenseNet121	Smoothed saliency maps; Grad-CAM	No	No
Beeche et al. [16]	CRVO; BRVO	Public datasets via GitHub (PU); STARE, USA (PU)	CFP	7,062	Classification	DL	NUN; ResNet; DenseNet; Inception-v3	GVM; CAM	STARE PU dataset (397 CFP images)	No
Chen et al. [17]	BRVO; CRVO; other diseases	Tianjin Medical University EyeHospital, China (PR); Kaggle Diabetic Retinopathy Detection dataset, China (PU)	CFP	8,600	Classification; segmentation (lesion)	DL	Inception-v3; DenseNet-121; Resnet-50; SEResNext-50 (class.) FCN-32s; DeepLab-v3; DANet; Lesion-Net-8s (segm.)	N/R	PR dataset (224 CFP images)	No
Dong et al. [18]	RVO; 9 other retinal diseases	Beijing Tongren Hospital, China (PR); iKang Health Care Centers, China (PR); Beijing Eye Study dataset, China (PR); Kailuan Eye Study dataset, China (PR)	CFP	328,760	Classification	DL	RAIDS	Grad-CAM	Beijing Eye Study and Kailuan Eye Study PR datasets (10,084 CFP images)	No
Gallardo et al. [19]	nAMD; RVO; DME	University Hospital of Bern, Switzerland (PR)	OCT	710 (eyes)	Prediction (anti-VEGF treatment need)	ML	Random Forest	Feature importance	No	No
Gu et al. [20]	RVO; 13 other retinal diseases	6 primary healthcare centers in Shanghai and Xinjiang, China (PR)		9,590	Classification	DL	Airdoc Retinal Artificial System (ARAS) - Inception-ResNet-V2; Yolo-V3; EfficientNet-B3	N/R	No	Yes
Huang et al. [21]	BRVO; CRVO	Second Affiliated Hospital of Zhejiang University, China (PR); Second Affiliated Hospital of Xi’an Jiaotong, China (PR); Poland dataset, Poland (PR); Linfen dataset, China (PR); Ningbo dataset, China (PR)	FA	4,028	Classification	DL	VGG-19; ResNet-50; Inception-v3	N/R	Poland, Linfenand Ningbo PR datasets (230 FA images)	No
Ji et al. [22]	CRVO; BRVO; MRVO; healthy	Affiliated Eye Hospital of Nanjing Medical University, China (PR); Shenzhen Eye Hospital of Jinan University, China (PR)	CFP	914	Classification	DL	Swin Transformer	N/R	No	No
Kang et al. [23]	BRVO; CRVO; DME; nAMD; mCNV	Chang Gung Memorial Hospital, Taiwan (PR); Linkou Medical Center, Taiwan (PR); Taipei and Keelung branches, Taiwan (PR)	CFP; OCT; FA/ICG	35,355	Classification; prediction (anti-VEGF treatment need)	DL	EfficientNetB4 (class., pred.); Cascade R-CNN (ROI extraction)	Grad-CAM	No	No
Lin et al. [24]	RVO; 13 retinal diseases	51 clinical settings across China (PR)	CFP	260,830	Classification	DL	IneptionResnetV2	N/R	PR dataset from 35 different clinical settings (18,136 CFP images)	No
Masayoshi et al. [25]	BRVO	Keio University Hospital of Tokyo, Japan (PR)	CFP; FA; synthetic FA	403 pairs (CFP+FA)	Segmentation (nonperfusion area)	DL	GAN; U-Net	N/R	No	No
Miao et al. [26]	BRVO	First Affiliated Hospital of Nanjing Medical University, China (PR)	CFP	274	Classification (need for laser treatment); segmentation (nonperfusion area)	DL	VGG (class., laser treatment need); U-Net (segm., NPA detection)	N/R	No	No
Nagasato et al. [27]	BRVO; healthy	Tsukazaki Hospital, Japan (PR); Tokushima University Hospital, Japan (PR)	UWF-CFP	466	Classification	DL	VGG-16; SVM	Grad-CAM	No	No
Tang et al. [28]	RVO	Shanxi Eye Hospital, China (PR)	FA	161	Segmentation (nonperfusion area)	DL	CE-Net; CE-Deform-Net; DeepLabv3+; U-Net	N/R	No	No
Wan et al. [29]	BRVO; CRVO; MRVO; healthy	Shenzhen Eye Hospital, China (PR)	CFP	805	Classification	DL	Swin Transformer; VGG-16; VGG-19; MobileNet-v2; ResNet-18; ResNet-50; WP-CNN-105; DenseNet-121	Grad-CAM	No	No
Wong et al. [30]	BRVO; CRVO; DR; healthy	RFMiD, India (PU); Kaggle dataset, China (PU)	CFP	875	Classification	ML	Google's AutoML	N/R	Kaggle PU dataset (210 CFP images)	No
Xu et al. [31]	BRVO; CRVO	Department of Ophthalmology, Qilu Hospital, Shandong University, China (PR)	OCT	1,166	Prediction (of short-term anti-VEGF response via synthetic OCT generation)	DL	GAN	N/R	No	No
Xu et al. [32]	CRVO; BRVO; MBRVO; healthy	Eye Hospital affiliated with Nanjing Medical University, China (PR)	CFP	501	Classification	DL	ResNet 18; ResNet 18+SE; ResNet 18+CBAM; ResNet 18+CA	N/R	No	No
Yeung et al. [33]	BRVO; healthy	Chang Gung Memorial Hospital, Keelung, Taiwan (PR)	OCTA	120 (eyes)	Classification (macular ischemia severity); segmentation	DL	U-Net (class., segm.)	N/R	No	No
Zhang et al. [34]	BRVO; CRVO	Shanxi Eye Hospital, China (PR)	CFP	297	Classification	DL	VGG-CAM; ResNet-34; Inception-v3; MobileNet	Grad-CAM	No	No
Zhang et al. [35]	BRVO; healthy	Yancheng Third People's Hospital, China (PR)	FA	670	Classification	ML	HLBP + SVM	N/R	No	No
Zhang et al. [36]	RVO; ME	The Affiliated Eye Hospital, Nanjing Medical University, China (PR)	OCTA	2,800	Prediction (of RVO-ME recurrence after anti-VEGF treatment)	DL	VGG-19; Resnet-50; GoogLeNet; Inception-v3	N/R	No	No
Zhao et al. [37]	BRVO; CRVO; DR; retinal vasculitis; healthy	Zhongsan Ophthalmic Center, China (PR); SEH, China (PR); FSPH, China (PR)	FA	24,316	Classification; segmentation	DL	ResNet152; U-Net-VGG16 (Ai-Doctor)	Heatmaps	SEH and FSPH PR datasets (3,996 FA images)	No

**Table 2:** AI performance metrics AI: artificial intelligence, anti-VEGF: anti-vascular endothelial growth factor, AUC: area under the curve, BRVO: branch retinal vein occlusion, CLAHE: contrast limited adaptive histogram equalization, CRVO: central retinal vein occlusion, DSC: Dice similarity coefficient, DTL: deep transfer learning, FSPH: Foshan Second People's Hospital, MAE: mean absolute error, IoU: Intersection over Union, MRVO: macular retinal vein occlusion, NPV: negative predictive value, NUN: neural understanding network, PPV: positive predictive value, RVO: retinal vein occlusion, SEH: Shenzen Eye Hospital, ZOC: Zhongsan Ophthalmic Center

Study	Performance (internal)	Performance (external)	Performance (clinical)
Abitbol et al. [15]	Accuracy: 88.4%; sensitivity: 78.7%; specificity: 91.0%; precision: 77.2%; F1 score: 83.3%; AUC: 91.2%	-	-
Beeche et al. [16]	NUN (best model): accuracy: 0.911 (±0.007); sensitivity: 0.983 (±0.010); specificity: 0.803 (±0.005); precision: 0.881 (±(0.003); F1 score: 0.911 (±0.007); AUC (micro): 0.973 (±0.003); AUC (macro): 0.975 (±0.003); AUC (BRVO): 0.961 (±0.010); AUC (CRVO): 0.967 (±0.006)	NUN without transfer learning: AUC (micro): 0.900 (±0.018); AUC (macro): 0.897 (±0.021); AUC (BRVO): 0.934 (±0.013); AUC (CRVO): 0.898 (±0.057)	-
Chen et al. [17]	Inception-v3 (best model): BRVO: sensitivity: 1.00 (0.94-1.00); specificity: 1.00 (1.00-1.00); F1 score: 1.00; AUC: 1.00 (1.00-1.00); CRVO: sensitivity: 0.94 (0.81-0.99); specificity: 1.00 (0.99-1.00); F1 score: 0.97; AUC: 1.00 (0.99-1.00); Mean: sensitivity: 0.93; specificity: 0.99; F1 score: 0.95; AUC: 0.99	Inception-v3 (best model): BRVO: sensitivity: 0.80 (0.52-0.96); specificity: 0.98 (0.95-0.99); F1 score: 0.88; AUC: 0.95 (0.87-1.03); CRVO: sensitivity: 0.92 (0.62-1.00); specificity: 0.98 (0.95-0.99); F1 score: 0.95; AUC: 0.99 (0.94-1.03); Mean: sensitivity: 0.81; specificity: 0.90; F1 score: 0.85; AUC: 0.91	-
Dong et al. [18]	Accuracy (ALL): 95.3%-99.9%; accuracy (RVO): 0.974 (0.973-0.975); sensitivity (ALL): 89.8% (95% CI, 89.5%-90.1%)	Sensitivity (ALL-RAIDS): 91.7% (95% CI: 90.6%-92.8%); sensitivity (certified ophthalmologists): 83.7%; sensitivity (junior retinal specialists): 86.4%; sensitivity (senior retinal specialists): 88.5%	-
Gallardo et al. [19]	AUC (RVO/DME): 0.76 (for low demand); AUC (RVO/DME): 0.78 (for high demand)	-	-
Gu et al. [20]	-	-	RVO: accuracy: 0.99 (95% CI: 0.99-1.00); sensitivity: 0.79 (95% CI: 0.67-0.89); specificity: 1.00 (95% CI: 0.99-1.00); PPV: 0.82 (95% CI: 0.70-0.91); NPV: 1.00 (95% CI: 0.99-1.00)
Huang et al. [21]	Inception-v3 diagnosis (best model): accuracy: 0.8922; recall: 0.8826; precision: 0.8826; F1 score: 0.8849	Inception-v3 Diagnosis (best model): Poland dataset: accuracy: 0.3895; recall: 0.3849; precision: 0.2853; F1 score: 0.2992; Linfen dataset: accuracy: 0.8462; recall: 0.6755; precision: 0.6883; F1 score: 0.6804; Ningbo dataset: accuracy: 0.8280; recall: 0.8250; Precision: 0.8041; F1 score: 0.7951	-
Ji et al. [22]	BRVO: accuracy: 0.957; sensitivity: 0.917; specificity: 0.982; precision: 0.971; F1 score: 0.943; CRVO: accuracy: 0.978; sensitivity: 0.955; specificity: 0.986; precision: 0.955; F1 score: 0.955; MRVO: accuracy: 0.978; sensitivity: 1.000; specificity 0.976; precision: 0.800; F1 score: 0.887; Normal: accuracy: 1.000; sensitivity: 1.000; specificity: 1.000; precision: 1.000; F1 score: 1.000	-	-
Kang et al. [23]	Classification (diagnosis): BRVO: accuracy: 0.977; sensitivity: 0.690; specificity: 0.997; CRVO: accuracy: 0.977; sensitivity: 0.769; specificity: 0.983; Prediction (need for anti-VEGF treatment): accuracy: 0.930; sensitivity: 0.904; specificity: 0.945; Control group (no treatment needed): accuracy: 0.984; sensitivity: 0.971; specificity: 0.985	-	-
Lin et al. [24]	RVO: sensitivity: 0.945; specificity: 0.905; AUC: 0.962 (0.959-0.966)	RVO (tertiary hospital set): AUC: 0.948 (95% CI: 0.940-0.956)	-
Masayoshi et al. [25]	Dice score: 0.82	-	-
Miao et al. [26]	Classification: accuracy: 0.79 ± 0.02; recall (sensitivity): 0.75 ± 0.08; precision: 0.80 ± 0.07; AUC: 0.82 ± 0.03; segmentation: accuracy: 0.89 ± 0.02; recall: 0.74 ± 0.05; precision: 0.87 ± 0.02; F1-score: 0.80 ± 0.03; AUC: 0.96 ± 0.02	-	-
Nagasato et al. [27]	VGG-16: sensitivity: 94.0% (93.8%-98.8%); specificity: 97.0% (89.7%-96.4%); PPV: 96.5% (94.3%-98.7%); NPV: 93.2% (90.5%-96%); AUC: 0.976 (0.960-0.993)	-	-
Tang et al. [28]	Dice (CE-Net): 0.928 ± 0.064 (with CLAHE); Dice (CE-deform-Net): 0.928 ± 0.066 (with CLAHE)	-	-
Wan et al. [29]	Swin Transformer (best model): BRVO: accuracy: 98.88 ± 0.080; sensitivity: 98.55 ± 0.056; specificity: 99.04 ± 0.041; precision: 98.56 ± 0.066; F1: 96.56 ± 0.068; CRVO: accuracy: 99.98 ± 0.015; sensitivity: 99.97 ± 0.016; specificity: 99.99 ± 0.006; precision: 99.73 ± 0.062; F1: 99.99 ± 0.006; MRVO: accuracy: 94.49 ± 0.094; sensitivity: 93.89 ± 0.095; specificity: 99.98 ± 0.017; precision: 99.97 ± 0.026; F1: 96.81 ± 0.084; Normal accuracy: 99.42 ± 0.012; sensitivity: 99.99 ± 0.0001; specificity: 99.12 ± 0.031; precision: 98.19 ± 0.065; F1: 99.19 ± 0.085	-	-
Wong et al. [30]	BRVO: accuracy: 96.51%; sensitivity: 71.43%; recall: 71.4%; specificity: 98.73%; precision: 83.3%; CRVO: accuracy: 98.81%; sensitivity: 66.67%; recall: 66.7%; specificity: 100%; precision: 100%	BRVO: accuracy: 96.81%; sensitivity: 90.91%; recall: 90.9%; specificity: 98.61%; precision: 95.2%; F1: 0.93; CRVO: accuracy: 98.38%; sensitivity: 95.45%; recall: 95.5%; specificity: 98.77%; precision: 91.3%	-
Xu et al. [31]	MAE (overall): 26.33 ± 15.81; MAE (BRVO classification): 24.21 ± 14.82; MAE (CRVO classification): 28.55 ± 17.32	-	-
Xu et al. [32]	ResNet 18+CA (best model): BRVO: accuracy: 0.9464; sensitivity: 0.8333; specificity: 0.9091; F1: 0.8696; CRVO: accuracy: 0.9821; sensitivity: 1.000; specificity: 0.8750; F1: 0.9333; MRVO: accuracy: 0.9643; sensitivity: 0.8333; specificity: 0.8333; F1: 0.8333	-	-
Yeung et al. [33]	Accuracy: 0.865; sensitivity: 0.757; specificity: 0.916; precision: 0.813; F1: 0.781	-	-
Zhang et al. [34]	VGG-CAM (best model): BRVO: sensitivity: 0.94; specificity: 0.99; AUC: 0.99; Kappa: 0.97; CRVO: sensitivity: 0.99; specificity: 0.96; AUC: 0.99; Kappa: 0.88	-	-
Zhang et al. [35]	Accuracy (mean): 96.1%	-	-
Zhang et al. [36]	VGG-19 (best model): DTL: accuracy: 0.913; sensitivity (recall): 0.922; specificity: 0.902; precision: 0.922; F1: 0.922; AUC: 0.968 (95% CI: 0.943-0.994); Fusion: accuracy: 0.935; sensitivity (recall): 0.935; specificity: 0.934; precision: 0.947; F1: 0.941; AUC: 0.972 (95% CI: 0.946-0.997)	-	-
Zhao et al. [37]	BRVO classification (ZOC): accuracy: 0.932 (0.915-0.949); recall: 0.930 (0.912-0.948); precision: 0.970 (0.958-0.982); AUC: 0.985 (0.977-0.993); BRVO segmentation (ZOC): DSC: 94.4 (90.4-98.4); IoU: 89.4 (84.0-94.8); F1: 92.0 (87.3-96.7); CRVO segmentation (ZOC): DSC: 89.2 (83.4-95.0); IoU: 80.6 (73.2-88.0); F1: 84.0 (77.2-90.8); KAII (BRVO - laser therapy decision threshold): sensitivity: 86.24%; specificity: 93.83%; AUC: 0.955 (0.933-0.976)	BRVO classification (SEH): accuracy: 0.921 (0.888-0.954); recall: 0.920 (0.887-0.953); precision: 0.950 (0.923-0.977); AUC: 0.963 (0.940-0.986); BRVO classification (FSPH): accuracy: 0.933 (0.909-0.957); recall: 0.930 (0.906-0.954); precision: 0.960 (0.942-0.978); AUC: 0.972 (0.956-0.988); BRVO segmentation (SEH): DSC: 92.5 (87.9-97.1); IoU: 86.0 (80.0-92.0); F1: 89.4 (84.0-94.8); BRVO segmentation (FSPH): DSC: 91.7 (86.9-96.5); IoU: 84.7 (78.4-91.0); F1: 88.3 (82.7-93.9)	-

We observed that most studies (57%) used color fundus photographs (CFPs) or ultrawide-field color fundus photographs (UWF-CFPs) (9%) as the modality of choice, due to the fact that it is a noninvasive, reproducible, and easy-to-conduct examination. Twenty-six percent of the studies used FA as the imaging modality of choice. Two studies used OCT images [19,31], two studies used OCTA [33,36], and two studies employed a combination of modalities (CFP+OCT+FA/indocyanine green angiography and CFP+FA) [23,25]. Most studies that applied AI for the classification of RVO used CFP as the preferred modality. FA was mainly used in studies focusing on the segmentation of areas of nonperfusion and treatment prediction, while OCT and OCTA were used exclusively in studies on treatment prediction.

The number of images varied widely across studies, with dataset sizes ranging from 161 to 328,760 images. Since the various datasets differ in type and contain various data formats, it is impossible to make absolute numerical comparisons, only broad stratifications by size. The various dataset sizes used in the studies also fluctuated in type or data format. Only 30% of the studies used external validation datasets to test their AI algorithms, and one study included clinical validation datasets [20].

The vast majority of the studies (87%) used DL Algorithms, while only three used ML Algorithms [19,30,35]. Seventy-eight percent of studies used some type of CNN (one or more), mostly a subtype of Residual Network (ResNet). Two studies implemented Swin Transformer (Microsoft Research Asia, Beijing, China) [22,29], one of which used Swin Transformer in combination with CNN algorithms [29]. Two studies also used the support vector machine (SVM) algorithm [27,35], one in combination with a CNN [27] and one in combination with a hierarchical local binary pattern [35]. One study used an automated ML algorithm from Google, but did not specify the algorithm type [30].

More specifically, the most frequent AI algorithms used for classification purposes were Densely Connected Convolutional Network (DenseNet), ResNet, and Visual Geometry Group Network (VGG). These were used by 67% (12 out of 18) of classification studies, while from the six remaining studies, one used NUN [16], one used U-Net [33], two used Swin Transformer [22,29], and two used SVM algorithms [27,35]. Concerning segmentation, the vast majority (five out of six studies) used the U-Net AI algorithm either alone or in combination with other algorithms [25,26,28,33,37]. For predicting RVO treatment, each study used its own AI algorithms. Out of four studies [19,23,31,36], only one used more than one algorithm for prediction purposes [36].

Concerning internal validation metrics, accuracy ranged from 0.79 to 0.99, sensitivity from 0.67 to 1.00, specificity from 0.80 to 1.00, precision from 0.77 to 1.00, area under the curve (AUC) from 0.76 to 1.00, and F1 score from 0.78 to 1.00. Concerning external validation metrics, accuracy ranged from 0.39 to 0.98, sensitivity from 0.38 to approximately 0.93, specificity from 0.90 to 0.98, precision from 0.29 to 0.96, AUC from 0.90 to 0.99, and F1 scores from about 0.30 to 0.89. Multisite external testing showed heterogeneous performance across cohorts, for example, the Poland, Linfen, and Ningbo sets in Huang et al.'s study [21] and the cross-hospital evaluations in Zhao et al.'s study [37].

Segmentation studies reported Dice similarity coefficients from approximately 0.82-0.94 and Intersection over Union from approximately 0.81-0.89, while prediction studies used mean absolute error (MAE) for continuous targets and AUC or accuracy for categorical targets such as treatment need or recurrence. Among the four prediction studies, three reported AUC or accuracy [19,23,36], and one reported MAE [31].

Thirty-nine percent of studies used comprehensive XAI techniques, most of which (seven studies) used Grad-CAM [15,16,18,23,27,29,34]. From the studies that performed prediction tasks, two applied XAI techniques: one used Grad-CAM [23] and the other employed feature importance [19].

Discussion

To the best of our knowledge, this is the first review concerning the use of AI for the diagnosis (classification), segmentation, and treatment prediction of RVO. Based on the statistical results provided by the researchers, the majority of the studies (91%) were conducted after 2020. Most studies evaluating the classification/diagnosis of RVO with AI models used color fundus images as input data, whereas most studies evaluating RVO treatment used OCT images as input data. DL algorithms were used in most studies, with AI performance values varying between different studies.

In studies that included both internal and external validation, the internal testing metrics were consistently higher than those obtained from external or real-time validation. Specifically, for the AUC metric, the mean value for internal validation was 3.75% higher. The mean difference in sensitivity was 9%, while specificity showed the smallest mean difference at 1.73%, both favoring internal testing. In one study [30], the external testing sensitivity was reported to be significantly higher than the internal testing value (25.9% higher); however, the overall mean still indicated superior internal testing metrics. The mean differences for accuracy and precision were 10.08% and 16.31%, respectively, with precision showing the largest difference between internal and external validation. The mean difference for the F1 score was 13.96%.

In the study by Huang et al. [21], a substantial discrepancy was observed between internal and external validation metrics, attributed to one external validation group reporting very low results. The authors did not provide an explanation for this finding. Studies employing Swin Transformer models reported the best diagnostic and classification performance for RVO based on internal validation metrics [22,29]. However, these results cannot be safely compared with other studies, as they did not include external validation data. Overall, all models demonstrated lower specificity than their near-perfect sensitivity.

These patterns are consistent with a domain shift that arises from differences in camera vendors, acquisition protocols, case mix, disease prevalence, and image quality. Internal testing, therefore, risks overestimating real-world performance. Practical mitigation includes multisite curation with balanced representation of devices and populations, vendor-stratified reporting, color and illumination harmonization, augmentation that reflects acquisition variability, and prespecified external evaluations with patient-level splits. Reporting per-site confusion matrices and explicit internal to external deltas would make transportability easier to judge.

Additionally, complexity occurs with the use of different statistical analysis models in comparing results. Also, image-capturing methods, image quality, and analysis techniques differ across various studies, resulting in some inconsistencies in findings.

A few studies leveraged UWF-CFP imaging for classification, which is attractive because peripheral findings carry clinical weight in RVO [15,27]. At the same time, UWF-CFP images introduce peripheral distortion and uneven illumination that can mislead saliency methods and classifiers and that may differ across devices. Model development with ultrawide-field data should, therefore, include distortion correction, peripheral intensity normalization, and careful definition of valid regions for analysis. Reporting results both on full ultrawide-field views and on centrally cropped fields would clarify the added value of the extended field of view.

Most of the studies (87%) were conducted in Asian countries, namely China, Taiwan, and Japan, which might influence the generalizability of the findings. The AI models and parameters used vary between different studies, making it difficult to compare them directly.

Most models used a single imaging modality, with CFP dominating classification tasks and FA featuring prominently in segmentation and treatment prediction. Only a small number of studies combined modalities, typically as limited pairs such as fundus with angiography or fundus with angiography and OCT [23,25]. There is a clear opportunity for multimodal fusion that brings together structural and perfusion information. Combining OCT or OCTA with angiography can reduce missed ischemia and support more reliable prediction of treatment response. Late fusion and attention-based fusion offer tractable approaches that preserve interpretability while exploiting complementary signals.

Ground-truth creation relied on expert annotation, yet methods for managing variability among graders were usually not described. Without data on agreement, adjudication, or label curation, it is difficult to understand how label noise influenced learning and evaluation, especially for subtle distinctions between branch and central occlusions and for delineating nonperfusion areas. Future work would benefit from multigrader protocols with consensus or arbitration, routine reporting of interrater statistics, formal label audits, and explicit handling of uncertain or borderline cases. Clear documentation of annotation tools and instructions would also improve reproducibility and interpretability.

Beyond offline evaluation, prospective assessment embedded in routine care is needed to understand workflow impact, safety, and clinician acceptance. With clinical validation reported only once [20], current evidence does not address how these systems influence referral accuracy, time to decision, or reading room workload. Useful designs include silent shadow deployments that record model outputs without altering care, followed by controlled rollouts that measure operational and clinical endpoints. Prespecification of operating points and calibration monitoring can support safe triage, while site-level analyses can reveal where models help and where they fail.

XAI appeared in a substantial minority of studies, most often as Grad-CAM visualizations, yet it was usually presented as an illustrative figure rather than a tool for systematic audit. A more rigorous approach would move from qualitative heat maps to quantitative tests. Two practical checks are whether highlighted regions align with ophthalmologist-defined structures, such as hemorrhages, cotton wool spots, and areas of nonperfusion, and whether heat concentrates on clinically causal features rather than on artifacts or spurious cues. Error analysis that groups false-positives and false-negatives by saliency patterns can reveal consistent failure modes and guide targeted data curation. When prediction models rely on feature importance, influential variables should be related to known pathophysiology and tested for stability across sites and time. Publishing code and prespecified quality control procedures for saliency would make explainability actionable and reproducible.

Ethical considerations, like patient privacy and data security, must be addressed to ensure the responsible use of AI. It is important to ensure the protection of patients’ data as AI applications require access to sensitive health information. Data anonymization and secure storage can prevent unauthorized access or data breaches. Moreover, transparent algorithms, explainability, and clear usage policies are essential to build trust among patients and healthcare providers. Finally, implementing regulations such as the AI Act [38] can help establish standards and guidelines to safeguard these aspects.

While AI models can assist ophthalmologists in the diagnosis, segmentation, and treatment prediction of RVO, they cannot yet replace expert judgment. Clinical decisions, particularly those involving treatment, should not be based solely on AI outputs but should be verified by ophthalmologists to ensure patient safety and responsible implementation.

## Conclusions

AI has demonstrated considerable promise in the diagnosis, segmentation, and prediction of treatment for RVO. Most studies have focused on classification tasks using CFPs, with DL, particularly CNNs, being the predominant approach. However, model performance remains variable, and internal validation results often overestimate real-world accuracy due to limited external and clinical validation. The generalizability of current findings is further constrained by geographical concentration, dataset heterogeneity, and inconsistent methodological reporting.

Future research should prioritize the development of multimodal and externally validated models trained on diverse, well-annotated datasets. Standardized reporting of performance metrics, inclusion of multi-grader consensus in ground truth creation, and incorporation of explainable AI methods will enhance transparency and clinical applicability. Ethical and regulatory considerations, especially those related to data privacy, accountability, and compliance with frameworks such as the European Union AI Act, are essential to guide responsible adoption. While AI systems can serve as valuable decision-support tools for ophthalmologists, they should complement rather than replace clinical expertise. Their integration into ophthalmic practice must be guided by rigorous validation, transparency, and ongoing human oversight to ensure patient safety and trustworthy implementation.
